# Design of Entry Detection Method for Top-Bounded Spaces Using GPS SNR and Spatial Characteristics for Seamless Positioning in Logistics Facilities

**DOI:** 10.3390/s20236864

**Published:** 2020-11-30

**Authors:** Kenichi Tabata, Madoka Nakajima, Naohiko Kohtake

**Affiliations:** Graduate School of System Design and Management, Keio University, Kanagawa 223-8526, Japan; madoka.nakajima@sdm.keio.ac.jp (M.N.); kohtake@sdm.keio.ac.jp (N.K.)

**Keywords:** top-bounded space, GPS SNR, seamless positioning

## Abstract

With the widespread use of indoor positioning technology, various services based on this technology are beginning to be offered to consumers and industrial applications. In the case of logistics facilities, in addition to indoor and outdoor spaces, there are top-bounded spaces (TBSs): elongated areas that are covered with roofs or eaves on the upper parts of buildings. The sides of such spaces are open, and workers and forklifts work in these areas. Only a few studies have been conducted on positioning methods for this unusual environment, and the way by which Signal-to-Noise Ratio (SNR) of Global Positioning System (GPS) changes with the stay in TBSs is unclear. Therefore, we conducted preliminary experiments and confirmed that TBS dwellings are difficult to stably detect with existing methods due to the combination of satellites with variable and unchanged SNRs. In this study, we designed a simple processing flow for selecting satellites with high probabilities of changing SNRs by using the spatial characteristics of TBSs as parameters (height, depth, and side opening orientation). We propose a method to detect the stay in TBSs using the SNR change rates of the selected satellites. As a result of evaluation experiments with three TBSs, we successfully detected the stay in TBSs with about 30% higher probability than those of an existing method.

## 1. Introduction

Indoor positioning technology has advanced significantly in the last decade due to the availability of inexpensive and easy-to-use sensors for indoor positioning, such as smartphones and iBeacon [1,2]. In addition, high-accuracy positioning systems are now available and beginning to be used in hospitals, factories, and logistics facilities [3,4]. For consumers, indoor positioning applications are available at airports and museums; in industrial applications, they are used to improve work efficiency by tracking moving objects, such as workers and forklifts in manufacturing and logistics [5,6,7,8]. Therefore, the use of indoor positioning systems in the manufacturing and logistics industries is expected to increase in the future, and the market size is expected to grow [9]. In addition, indoor positioning can be seamlessly connected to outdoor positioning using Global Positioning System (GPS) to further improve its availability. In industrial applications, continuous tracking of workers and forklifts indoors and outdoors in large factory and logistics facilities, and continuous tracking of trucks, containers, and shipments from outside to inside logistics facilities will be possible in the logistics industry [10]. 

In large-scale logistics facilities, there are spaces that are covered with eaves or roofs on top and walls on one long side, where workers and forklifts work to carry in and store loads from large trucks. These spaces—the top is covered, and the sides are not completely closed—are called top-bounded spaces (TBS) [11]. Since the upper part of this space is covered, the accuracy of GPS positioning is expected to decrease, so an indoor positioning environment should be constructed. However, it is difficult to apply the indoor-outdoor (IO) detection method for indoor and outdoor spatial detection because the sides are open and GPS signals can be received to some extent in such an unusual environment [12]. If the system cannot detect the presence of workers and forklifts in a TBS, it will make wrong decisions as to whether to use outdoor positioning or indoor positioning when calculating its own position. As a result, the movement of workers and forklifts in the TBS will not be tracked correctly. Some studies have proposed IO detection methods based on the change rates of Global Navigation Satellite System (GNSS) Signal-to-Noise Ratio (SNR) with entry to TBSs, but the applicability of these methods to TBSs has not been clarified because the range of the target space has not been quantified [13,14].

Therefore, in this study, we first extracted a space with similar spatial characteristics to the TBS that exist in logistics facilities, and we confirmed the applicability of an existing method by conducting preliminary experiments. Our results confirmed that it is difficult to stably detect entry into TBSs using the existing method. The reason may be the lack of consideration for the constant change in satellite configuration and the differences between the spatial characteristics of targeted TBSs. In addition, the changes in SNR of individual GPS satellites show that satellites can be classified into two groups: those whose SNR values change significantly as they enter and exit a TBS and those whose SNR values do not change significantly as they enter and exit a TBS. For stable detection of the stay in a TBS, the detection method must take into account two things: the constantly changing arrangement of satellites and the spatial characteristics of the TBS, which vary between spaces. There are many methods of detecting the attenuation of GPS signals due to the influence of buildings. Such attenuation is based on the relationship between the positioning of satellites and the current position of receiving device, but the construction of a precise three-dimensional (3D) model that considers TBSs is a problem in terms of cost. Therefore, we designed a method to easily identify satellites needed for detection by using three spatial characteristics of TBSs as parameters: height, depth, and side opening orientation. Three different TBSs were extracted from the preliminary experiments and evaluated to verify the effectiveness of the designed detection method. The results confirmed that the proposed method detects entry into and exit from TBSs with a higher probability than those of the existing method. In addition, by conducting experiments with different smartphone models, we detected entry into and exit from the TBSs in the same way, regardless of phone model.

Our proposed method specializes in detecting entry into and exit from TBSs without position calculation. In the application of this technique, it is assumed that GPS positioning is used outdoors, and indoor positioning environments are established for indoor and TBS environments. By incorporating the proposed method as a component in seamless positioning in logistics facilities, we aim to support the construction of a simple and inexpensive seamless positioning environment that also considers TBSs.

In addition, the shapes of TBSs in real space are so varied that it is difficult to validate the effectiveness of the proposed method for all patterns of TBSs. In this study, we target a TBS with an elongated rectangular shape when viewed from above, with one side of the long side covered by a wall. For example, TBSs with a square shape, TBSs that are open on all four sides, and TBSs that are not rectangular in shape are out of scope in this study.

The remainder of this paper is organized as follows: Section 2 provides an overview of studies relevant to our proposed method. In Section 3, two locations with spatial characteristics similar to those of TBSs in logistics facilities were selected, and preliminary experiments were conducted. An overview of the preliminary experiments and the results is also presented. In Section 4, we designed the method of detecting entry into and exit from TBSs based on the results of the preliminary experiments. Three different TBSs were selected from the preliminary experiments, and we conducted evaluation experiments in Section 5 to confirm the effectiveness of the proposed method. Based on the results of the evaluation experiments, a discussion is presented in Section 6. Finally, Section 7 summarizes this study.

## 2. Related Work

### 2.1. IO Detection Method

Determining whether an object is outdoors or indoors is an important elemental process in seamless positioning. This technology is called IO detection or handover [15]. Various approaches have been studied to achieve accurate IO detection; these methods either combine information from various sensors that can be acquired by smartphones or use only GPS signals [16,17,18]. For methods that use only GPS signals, many studies focused on the change in the GPS SNR when entering an indoor area from outdoors. The authors in [19] noted that, when entering a building from outdoors, satellites with high-elevation angles have a large attenuation of the SNR just before entering the building, whereas satellites with low elevation angles have less attenuation. Then, they proposed a method for detecting entry into a building by selecting satellites with high-elevation angles among available satellites and tracking the change in the SNR value. A method for detecting outdoor-to-indoor entry has also been proposed using three parameters: the number of satellites that can be acquired, Dilution of Precision (DOP), and SNR [20]. Both methods are effective for movement between outdoor and indoor spaces, but their effectiveness for areas such as TBSs is unclear. A method that uses machine learning based on location information that can be acquired to detect indoor/outdoor decisions with almost 100% accuracy has also been proposed, but the applicability of this method to spaces such as TBSs has not been clarified [21].

### 2.2. Seamless Positioning Method Considering TBS

Although a number of indoor/outdoor seamless positioning methods have been proposed, only a few studies have been conducted on seamless positioning methods that also consider TBSs [22]. The authors in [13] defined corridor-shaped TBSs as semi-outdoor areas and proposed a seamless positioning method that is applicable in three spatial contexts: outdoor, semi-outdoor, and indoor. Their method of detecting entry into TBSs is based on a 20% reduction in the average obtainable GPS SNRs compared with that for outdoor spaces. However, it is not clear whether their proposed method can detect entry into TBSs, since the target spaces in their study and our study have different spatial characteristics. Similar to that in [13], the seamless positioning method proposed in [17] can be applied within three spatial contexts, namely, outdoor, semi-outdoor, and indoor, but uses smartphones’ optical sensors, geomagnetic sensors, and base station information for detection. Their method is difficult to apply to detect workers in distribution facilities, as the use of optical sensors places significant restrictions on the holding of the smartphone. The author in [14] defined the so-called typical intermediate: a space where the top surface is covered by a building and at least one side of the area is open; then, they proposed a method for detecting four spatial classifications using GPS SNR: open-sky, urban, intermediate, and indoor. Their method uses the number of available satellites and the SNR as parameters, and it aims to improve the detection rate by targeting satellites with SNR values of 25 dB-Hz or higher. They reported high detection rates for open-sky, urban, and indoor, but that of intermediate was not high.

### 2.3. LOS/NLOS Detection Method Using GNSS SNR

Changes in GNSS SNR values were used in [13,14] to detect entry into indoor and TBS areas. This change in value is caused by the inability of the receiving device to receive GNSS signals directly due to the influence of buildings and other shielding (this condition is called non-line-of-sight (NLOS)); the SNR under this condition is lower than that where GNSS signals can be received directly (this condition is called line-of-sight (LOS)) [23]. LOS/NLOS is typically determined using a 3D model of the building. This method calculates the LOS/NLOS ratio of the SNR of the satellite using information that is previously retained as fingerprints of the satellite’s visibility status at each position by matching the satellite’s position with a 3D model. Then, it aims to improve positioning accuracy in urban areas by adopting only satellites that are estimated to be LOS [24]. Although the use of 3D models makes it possible to predict LOS/NLOS to some extent, the construction of a 3D model that considers the shapes of eaves requires modeling at or above the Level-of-Detail (LOD) 2 level, as defined by City Geography Markup Language (CityGML), which is a challenge in terms of model construction cost [25].

## 3. Preliminary Experiments

### 3.1. Overview of Preliminary Experiments

**Figure 1:**
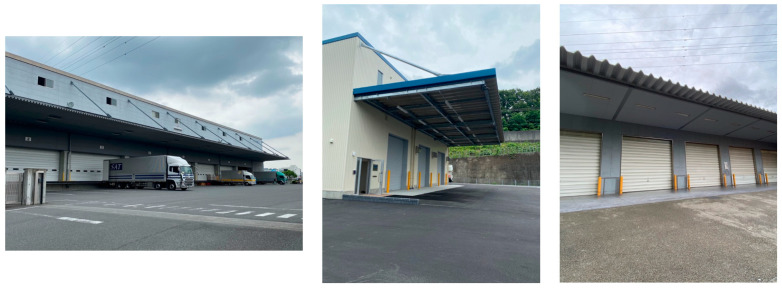
Typical top-bounded spaces (TBSs) in logistics facilities.

**Figure 2:**
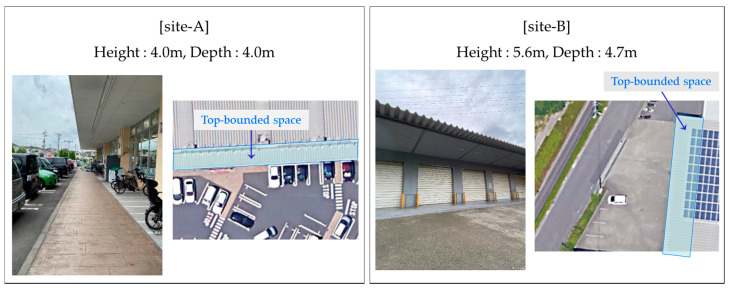
Overview of preliminary experiment sites A and B.

The targeted TBS is a space covered by a large eave, which is commonly seen in logistics facilities, as shown in Figure 1. The space is rectangular when viewed from directly above, with a wall on one long side and no wall on the other sides. As shown in Section 2, seamless positioning methods considering TBSs have been used in some studies, but it is unclear whether these existing methods can be applied to TBSs, as shown in Figure 1, due to the different spatial characteristics between such structures. In addition, the way by which the GPS signals acquired in this unusual environment change has not been sufficiently clarified. To confirm the applicability of an existing method to TBSs and the change in GPS signals during entry into TBSs, we conducted preliminary experiments at two locations with similar characteristics, as shown in Figure 1. The space in which the preliminary experiments were conducted is shown in Figure 2. The target spaces were TBSs with a height and depth of more than 4 m, as the height of large trucks used for unloading and loading at logistics facilities is just under 4 m. The height and depth of the TBSs were measured with a laser rangefinder to see if these conditions were satisfied.

**Figure 3:**
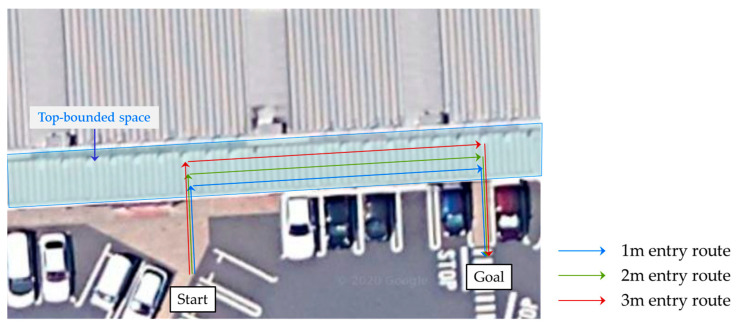
Walking route of preliminary experiment at site-A.

**Figure 4:**
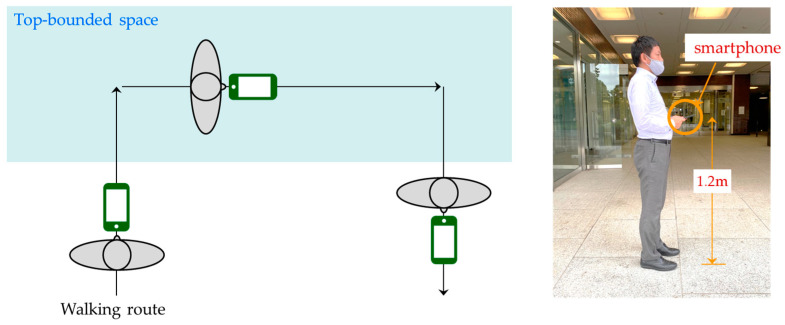
Smart phone holding condition during the experiment.

In the preliminary experiments, a tester entered each TBS at a speed of about 1 m/s, walked about 20 m inside the TBS, and returned outside. During the walk, we measured the elevation angle, azimuth angle, and SNR values of each satellite every second in National Marine Electronics Association (NMEA) format using a smartphone application. The walking route in site-A is shown in Figure 3. Three routes, namely, 1 m, 2 m, and 3 m entering the TBS, were set up and measured three times to check the change in values depending on the degree of entry into the TBS. In addition, considering that the configuration of satellites constantly changes with the time of day, measurements were obtained in the morning and afternoon for each site. Each route was measured three times in the morning and three times in the afternoon; thus, there were 18 measurements per site, for a total of 36 measurements. A smartphone, model A (model name: Arrows m4, manufacturer: Fujitsu, Japan, OS version: Android 7.1.1), was used as the measurement device, and the tester walked holding the smartphone 1.2 m from the ground; the smartphone was fixed horizontally with the screen facing upward. Figure 4 shows the holding condition of the smartphone during the experiment.

### 3.2. Preliminary Experiment Result

**Figure 5:**
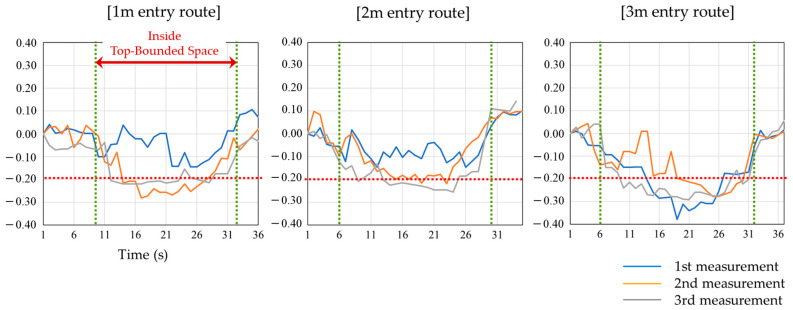
Change in average SNR for all satellites during walking experiment at site-A.

A graph showing the change in the overall satellite SNR average for each route during the afternoon experiment at site-A is shown in Figure 5. This graph is based on the average SNR of all satellites at the start of the walk and shows the percentage change relative to the base value for each second. The green vertical dotted lines indicate the times of entry and exit from the TBS, and the area enclosed by the vertical lines is the time of walking in the TBS. The red dotted line in the graph indicates the boundary of 20% decrease in the average value of the acquired SNR (dB-Hz). The method in [13] uses an SNR reduction of 20% or more relative to the outdoor walking SNR average as the threshold for entry into the corridor-shaped TBS, but this preliminary experiment shows various cases in which the SNR is decreased by 20% or more and cases in which it is not reduced. For example, on the 2-m entry route, the third measurement shows a reduction in SNR of more than 20% at most times, but the first and second measurements rarely show a reduction in SNR of more than 20%. Also, only the second measurement on the 3-m entry route shows a significant difference in the way the SNR changes from the other measurements.

**Figure 6:**
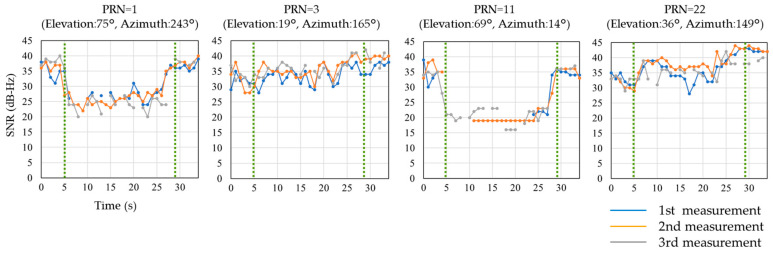
Change in SNR for each satellite during walking experiment at site-A.

Next, the changes in the SNR values for each of the major satellites are shown in Figure 6. The contents described above each graph are the pseudorandom noise (PRN) code, satellite elevation angle, and azimuth obtained from the NMEA format. As in Figure 5, the area surrounded by the green vertical dotted line is the walking time in the TBS, but the vertical axis shows the SNR value. The changes in the SNR values of each satellite show that the satellites with high-elevation angles show a significant decrease in SNR before and after the receiving device enters into the TBS, while the satellites with low elevation angles show no change in SNR after the receiving device enters. Moreover, the SNR value acquired by each measurement of the same route does not vary greatly, but the value sometimes cannot be acquired. For example, in Figure 6, there are cases where SNRs cannot be acquired for some satellites in the third measurement. With respect to PRN = 11, the SNR was not obtained for much of the time, even in the first measurement. This may be one of the reasons for the variation in the overall SNR average even for the same route. Given that the ratio of satellites with high and low elevation angles always changes because the configuration of satellites always changes, it is difficult for the existing approach (averaging the SNR over the entire satellite) to stably detect the approach to a TBS.

## 4. Design of TBS Entry Detection Method Using GPS SNR and Spatial Characteristics

### 4.1. Overview of Proposed Method

**Figure 7:**
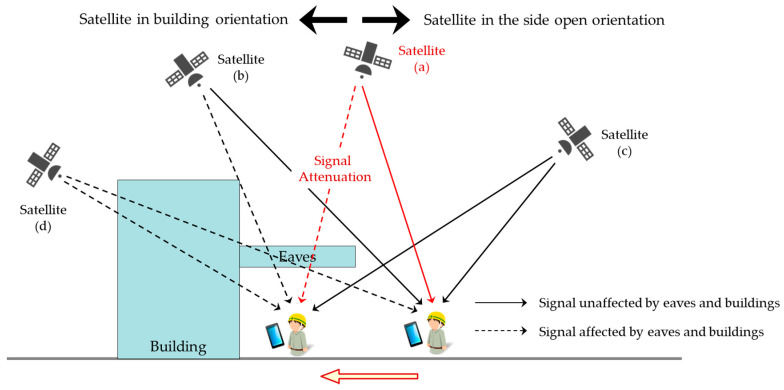
Relationship between satellite configuration and SNR attenuation considering top-bounded spaces (TBS).

Based on the results of preliminary experiments, we can estimate that TBS stays can be stably detected by monitoring the SNR changes of a satellite if the satellite whose SNR changes as the receiving device enters or exits the TBS can be identified. Although the configuration of satellites constantly changes, considering the presence of TBS, satellites in orbit can be classified into four groups, as shown in Figure 7. Satellite (a) is a satellite located at a high elevation angle and in the side open orientation. When outdoors, the receiving terminal can receive signals from this satellite without being affected by eaves and buildings, but once inside the TBS, it cannot receive sufficient signals due to the influence of eaves. Since Satellite (b) is located in the direction of the building, it is difficult to determine whether the attenuation of the SNR is caused by the eaves or the building, but the attenuation of the SNR does occur and can be used to detect the TBS stay. Satellites (c) and (d) are less likely to attenuate the SNR during the TBS stay due to their low elevation angles. Therefore, we can stably detect the stay in the TBS by identifying satellites belonging to Satellite (a) or (b) and monitoring the changes in the SNR.

### 4.2. Calculation of Entry Angle Thresholds and Classification of Satellites Using Spatial Characteristics

**Figure 8:**
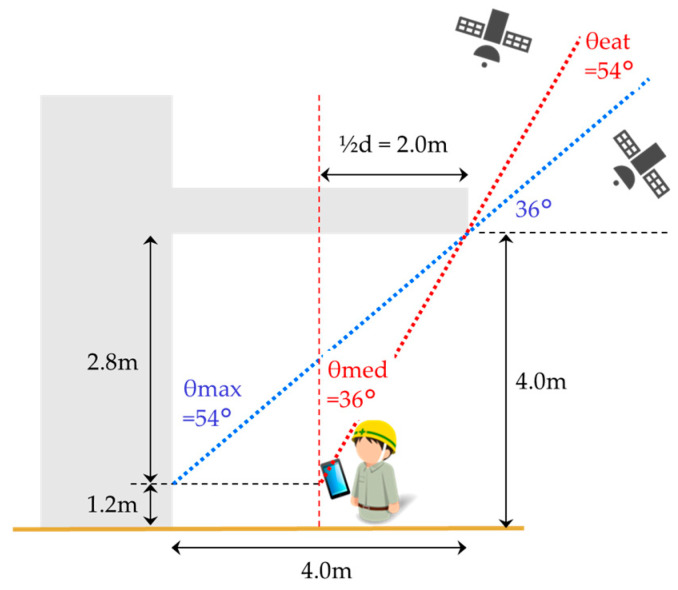
Calculation example of entry angle threshold at site-A.

**Figure 9:**
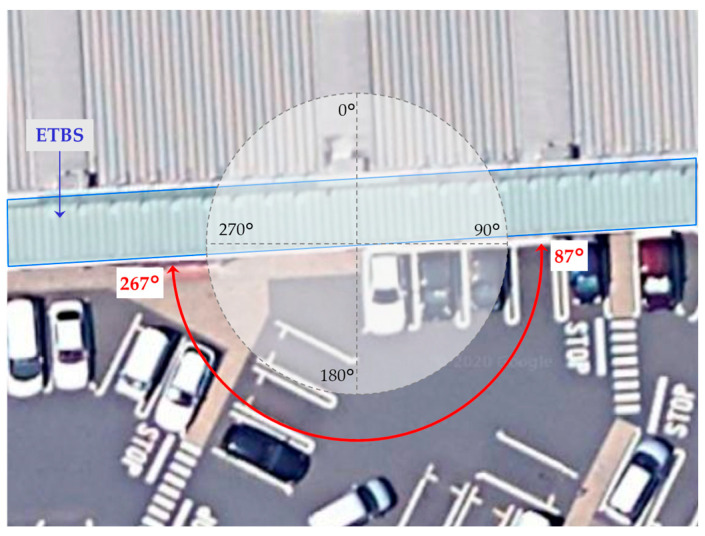
Example of side opening orientation at site-A.

It is difficult to extract the satellites to be monitored for TBS stay detection with a fixed threshold, since satellite configurations are constantly changing and TBS shapes vary. A precise 3D model would make it possible to determine whether the satellite is a target or not in relation to the position of the satellite. However, as mentioned in 2.3, this is problematic in terms of cost. Therefore, we designed a method to identify the satellites to be monitored in a simple way using the spatial characteristics of the TBSs whose stays need to be detected. By measuring the depth from the boundary to the wall and the height from the ground, we can quantify how deeply the TBS space can be entered with the following equation:

(1) $$\theta_{max}=\tan^{-1}\frac{D}{\left(H_{tbs}-H_{m}\right)}$$

where $\theta_{max}$ is the maximum entry angle, D is the depth of the TBS, $H_{tbs}$ is the height of the TBS, and $H_{m}$ is the holding height of the receiving device (smartphone). For example, in the case of site-A, the angle of entry to the deepest point is 54°, as shown in Figure 8. Thus, logically, when a worker with a smartphone enters the depth of the TBS, satellites with elevation angles higher than 36° will be affected by the eaves, and their SNRs will be attenuated. However, in practice, workers are expected to work at shallow angles of entry; that is, without going to the deepest point. To increase the possibility of detecting the TBS stay under such conditions, we set the entry angle based on one-half of the depth to extract the satellites affected by the eaves. In the case of site-A, this entry angle is 54°. In this study, this angle is defined as the entry angle threshold, which can be described by the following equation:

(2) $$\theta_{eat}=90-\theta_{med}$$

(3) $$\theta_{med}=\tan^{-1}\frac{\frac{1}{2}D}{\left(H_{tbs}-H_{m}\right)}$$

where $\theta_{eat}$ is the entry angle threshold and $\theta_{med}$ is the entry angle with respect to half of the depth. The side opening orientation can be estimated by using a geographic information system or Google Maps. In the case of site-A, 87°–267° is the side opening orientation, as shown in Figure 9.

Satellites in orbit can be classified into four groups, as shown in Table 1, according to two parameters: entry angle threshold and side opening orientation. The satellites in Group A are the highest-priority satellites for TBS entry detection. If there are no satellites in Group A due to satellite configuration, the next-highest-priority satellites will be the Group B satellites. In this study, we define the satellite extracted by this process as the monitoring target satellite.

**Figure 10:**
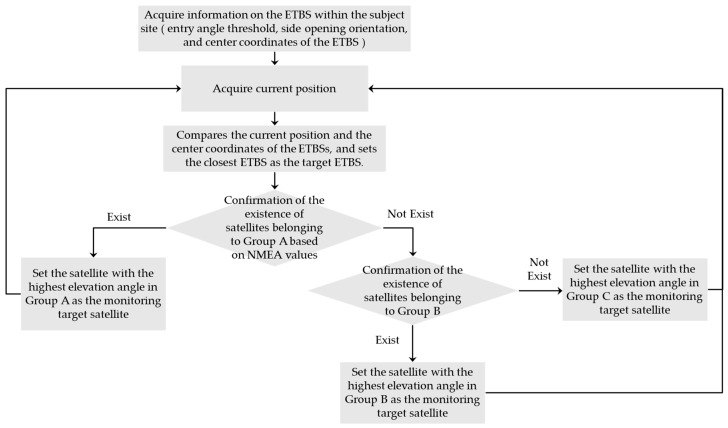
Process flow for selecting monitoring target satellite.

Figure 10 shows the process flow for selecting monitoring target satellite. The receiving device has prior information about the entry angle threshold, side opening orientation, and center coordinates of the TBS as parameters. Assuming that there are multiple TBSs in the target site, the nearest TBS, which is identified by comparing the center coordinates of each TBS from the current position of the receiving device, is determined as the target TBS. The receiving device checks the elevation and azimuth angles of each satellite, which are acquired in NMEA format, to see if any of these satellites belong to Group A. If there is a satellite belonging to Group A, if there is one satellite that belongs to it, that satellite is set as the monitoring target satellite. If more than one satellite belongs to Group A, the satellite with the highest elevation angle is set as the monitoring target satellite. If there are no Group A satellite, the satellite with the highest elevation angle among Group B satellites is set as the monitoring target satellite. If there are multiple satellites in the same group, the satellite with the highest elevation angle shall be set as the monitoring target. If there are no satellite in Group B in the satellite configuration, the satellite with the highest elevation angle among Group C satellites is set as the monitoring target satellite. However, in this case, the detection accuracy of the TBS stay is expected to be low because the monitoring target satellites have low elevation angles.

### 4.3. Detection of Entry into TBS Using SNR Attenuation

**Figure 11:**
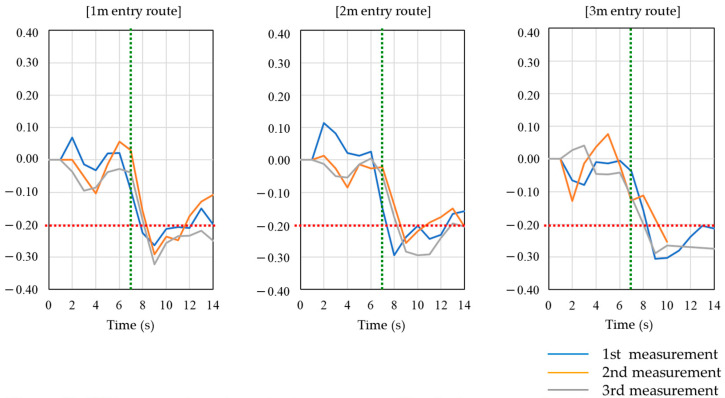
SNR attenuation of monitoring target satellite during entry of top-bounded spaces (TBS) in preliminary experiments.

**Figure 12:**
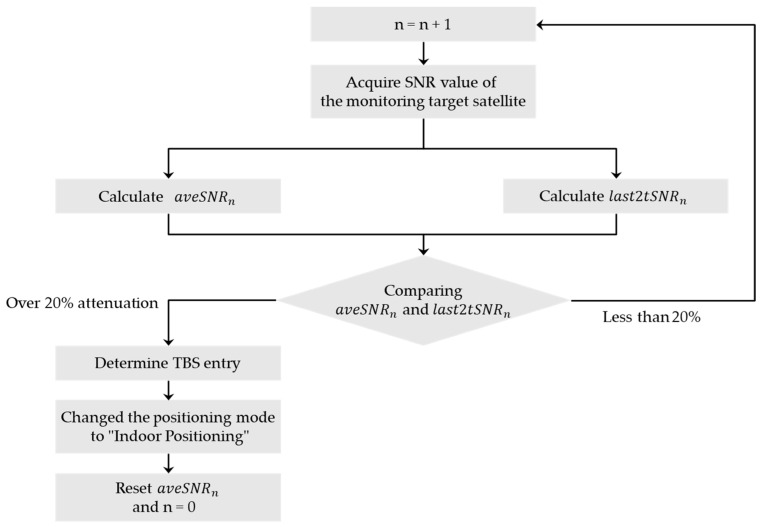
Determination flow for top-bounded spaces (TBS) entry.

Next, we designed a method for detecting TBS entry. Preliminary results show that the SNR values of some satellites decrease when the receiving device enter a TBS and increase when it exit the TBS. Similar results have been reported in other studies, as described in Section 2; a 20% attenuation of the average SNR for an overall satellite upon entering a corridor-shaped TBS was reported in [13]. Although not a case of entry into a TBS, the authors in [14] reported that a satellite’s SNR was attenuated by about 5 dB as the receiving device approached a building and was attenuated even more rapidly as it entered the building. Furthermore, a study reported that the SNR in outdoor spaces is 35–45 dB, whereas there is 25–35 dB for indoor spaces near windows due to 10–20 dB attenuation, as reported in [23]. Although these authors used different measurement devices, we can estimate that, in general, more than 20% of the attenuation in all cases occurs upon satellite entry into the TBS and indoors near windows. For the preliminary experiments in this study, Figure 11 and Table 2 show the extent to which the SNR of the satellite satisfying the conditions shown in 4.1 was attenuated upon satellite entry into the TBS. Figure 11 shows the SNR changes from outdoors to TBS entry of the selected satellites based on the processing flow shown in Figure 10 in the afternoon preliminary experiment at site-B. As in the other figures, the green vertical dotted line indicates the time of entry into the TBS; the left side of the vertical line is for the walk outdoors, and the right side is for the walking into the TBS. The vertical axis shows the extent to which the SNR average of the last two SNRs of the monitoring target satellite changes relative to the initial SNR average. The red dotted line indicates 20% attenuation. The rate of change of SNR at n times of data acquisition, used in this graph, can be expressed by the following equation:

(4) $$\delta_{n}\;=\;\frac{last2tSNR_{n}}{aveSNR_{n}}-1$$

(5) $$last2tSNR_{n}=\;\frac{\sum_{i=n-1}^{n}mtsSNR_{i}}{2}$$

(6) $$aveSNR_{n}=\;\frac{\sum_{i=1}^{n-2}mtsSNR_{i}}{n-2}$$

where $\delta_{n}$ denotes the rate of the SNR change, $mtsSNR_{i}$ denotes the SNR value of the monitoring target satellite for the *i*-th time, $last2tSNR_{n}$ denotes the average of the SNR of the monitoring target satellite for the last two times, $aveSNR_{n}$ denotes the average of the SNR of the monitoring target satellite before the last two times. For example, when starting data acquisition outdoors, $\delta_{n}$ is around 0 because the SNR value does not attenuate. When the receiving device enters the TBS, $aveSNR_{n}$ does not change much yet, but the $last2tSNR_{n}$ changes significantly due to the attenuation. The value of $\delta_{n}$ decreases accordingly. We can find that the change rate is much lower once the receiving device enters TBS compared to when it was walking outdoors. When the threshold for entry detection is set as 20%, we can determine the entry into the TBS by tracking the change in SNR of the monitoring target satellite. Table 2 shows the SNR attenuation rates for the monitoring target satellite at the time of TBS entry for the 36 measurements in the preliminary experiments. The average attenuation is 26.3%, with 20.5% attenuation occurring even at the lowest attenuation. Based on these results, the process flow for detecting entry into the TBS is shown in Figure 12. When $last2tSNR_{n}$ is 20% or more attenuated than $aveSNR_{n}$, we determine that the receiving device has entered the TBS. Then, the positioning mode is changed from outdoor positioning to indoor positioning. By changing the positioning mode, we can calculate the current position using the indoor positioning environment built in the TBS.

The processing flow for detecting the exit from the TBS to the outdoors utilizes the increase in the SNR of the monitoring target satellite as it exits the TBS. As in the determination flow in Figure 12, if $last2tSNR_{n}$ is increased by 20% or more compared with $aveSNR_{n}$, the receiving device is determined to have exited the TBS. Then, the positioning mode is changed from indoor positioning to outdoor positioning. By changing the positioning mode, we can calculate the current position using outdoor positioning.

## 5. Evaluation

**Figure 13:**
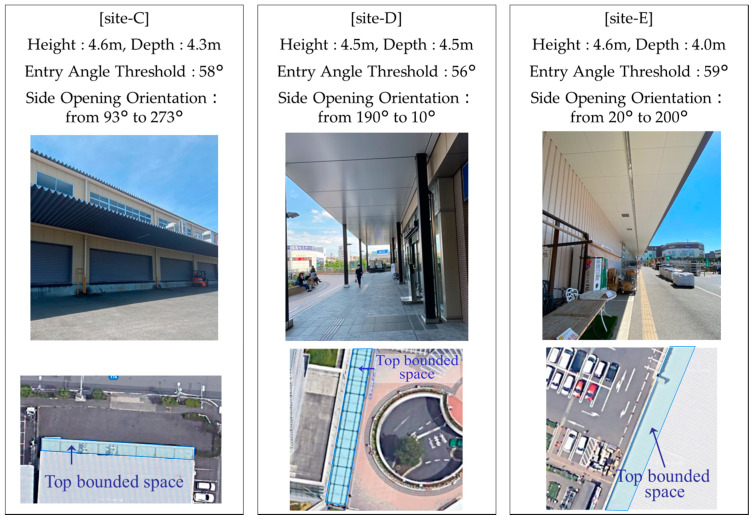
Overview of test sites C, D, E for evaluation experiments.

To confirm the effectiveness of the proposed method, we conducted evaluation experiments at three locations that had the same characteristics as those in the preliminary experiments. An overview of the evaluation experiment sites is shown in Figure 13. The measurement device was model A, which was the same as that in the preliminary experiments. The same measurement routes as those in the preliminary experiments was set up for each site in the evaluation experiments, and the tester walked at a speed of about 1 m/s with the smartphone held horizontally in front of their body at a height of 1.2 m above the ground. Then, the SNR, elevation angle, and azimuth angle for each satellite, recorded in NMEA format every second during the walk, were acquired using a smartphone application. For site-C and site-D, the 1 m entry, 2 m entry, and 3 m entry routes into the TBSs were measured three times each, and these measurements were made in the morning and afternoon for each site to consider the change in the satellite configuration with time. For site-E, we measured the 1 m entry and 2 m entry route three times, in the morning and afternoon. We obtained a total of 48 measurements.

### 5.1. Evaluation of Detection Accuracy of TBS Stay

**Figure 14:**
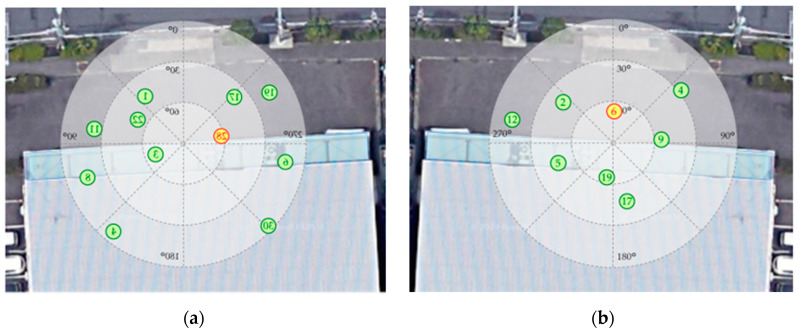
Example of satellite configuration during evaluation experiment at site-C: (**a**) morning experiment; (**b**) afternoon experiment.

To verify the detection accuracy of the proposed method, we recorded the time of the start of walking, the times of entering and exiting the TBS, and the time of the end of walking. The detection accuracy of the TBS stay was calculated by analyzing the recorded time and the change in the SNR of the monitoring target satellite, which was acquired by the smartphone application. As an example of the satellite configuration during the experiments, Figure 14 shows the satellite configuration during the morning and the afternoon for the 2 m entry experiments at site-C. The satellites indicated by the red dots are the satellites selected as monitoring target satellites based on the flow shown in Figure 9. The numbers in the dots indicate each satellite’s PRN code. From the 48 experiments, Group B satellites were selected as monitoring target satellite 6 times because Group A satellites do not exist.

**Figure 15:**
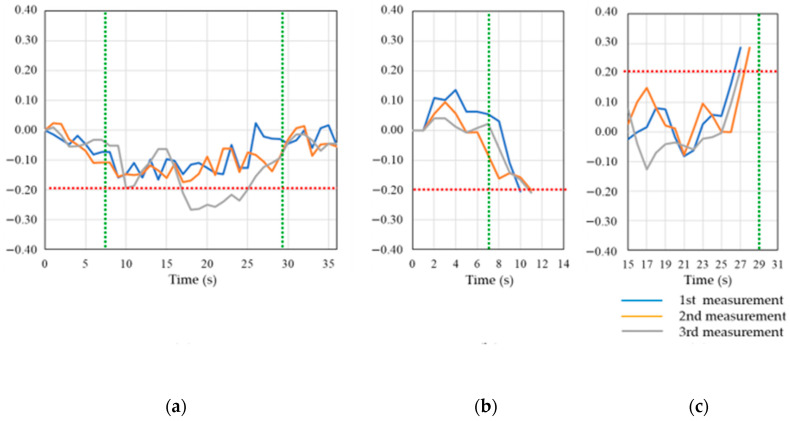
Comparison of SNR change between existing method and proposed method in evaluation experiment at site-C: (**a**) existing method; (**b**) proposed method (entry into TBS); (**c**) proposed method (exit from TBS).

To compare the detection accuracy of the proposed method with that of the method in [13], a graph describing the change in SNR during the 2 m entry experiment in the afternoon at site-C is shown in Figure 15. Figure 15a shows the change in the average SNR values for all the acquired satellites. The section bounded by the green vertical line is the walking section in the TBS, and the degree of change in the SNR average is shown on the vertical axis with respect to the start of the measurement. In the existing method, the criterion for judging the entry into the TBS is the point at which the average SNR value is attenuated by 20% compared with that of the outdoor space. Nonetheless, except for some sections of the third measurement, no attenuation exceeding 20% was observed. Therefore, when using existing methods, it is not possible to detect entry into the TBS in many cases. Based on the flow shown in Figure 12, Figure 15b,c shows the degree of change in the last two SNR averages relative to the overall SNR average of the monitoring target satellite. Figure 15b describes the change in SNR from outdoors to TBS entry, and Figure 15c describes the change in SNR from TBS to TBS exit to outdoors. Upon entry into the TBS, there is a three- or four-second delay from the actual entry, but the last two SNR averages show an attenuation of more than 20%; thus, entry into the TBS is detected. TBS exits are detected one or two seconds earlier than the actual exits, but an increase in the last two SNR averages of more than 20% occurred, and exits from the TBS are detected. As a result, all the entries into and exits from the TBS were detected by the proposed method in all three measurements. We determined whether the receiving device was staying in the TBS using the data measured every second and calculated the detection accuracy. As a result, the detection accuracy of the proposed method is 63.6% compared to 35.6% with the existing method. Thus, we can detect TBS stays more accurately than can the existing method. A table categorizing the measurement results according to the depth of entry is shown in Table 3. In the proposed method, the detection accuracy increases with the depth of entry, and the detection accuracy of the 3 m entry is 85.8%. Moreover, for 14 out of 18 times for the 2 m entry, the measurement is correctly detected more than half the time per measurement. This result indicates the number of times that a walk in TBS could be correctly determined over 11 s, e.g., if the walk in TBS for one measurement was 20 s. In other words, it shows that the stay at TBS is generally judged correctly, although there are times when a few seconds of discrepancy occurs during entry and exit from TBS and it is not judged correctly. In the case of the 3 m entry, all measurements are detected correctly.

### 5.2. Evaluation of Differences in Detection Accuracy by Receiving Devices

The GPS receivers in smartphones vary from model to model and differ in terms of performance. Different models may have varying SNR values acquired at the same location. Although the preliminary and evaluation experiments were conducted with the same model, we performed the same walking experiments with model B (model name: Pixel 3a, manufacturer: Google, United Status, OS version: Android 10) to confirm the effectiveness of the proposed method for different models, and we calculated its detection accuracy. During the experiment at site-D, we held models A and B in parallel and measured SNR values simultaneously.

Table 4 shows the measurement results for each model. Since the elevation and azimuth angles that can be acquired in NMEA format do not differ much between different models, the monitoring target satellites were the same. On the other hand, the acquired SNR values vary due to the differences in the phones’ GPS receivers, and there are variations in detection accuracy. The detection accuracy of model A is lowered because the attenuation does not often exceed the threshold value during the measurement of the 2 m entry. Model B shows stable attenuation upon TBS entry in all measurements. The detection accuracy is over 70% for both models, and we can confirm that the proposed method can be used with models other than model A.

## 6. Discussion

In this study, to stably detect stay in a TBS covered with large eaves and roofs, which is often seen in logistics facilities, we propose a method for detecting TBS stay by identifying a target satellite based on certain parameters. These parameters are spatial characteristics of the TBS: height, depth, and side opening orientation. The change in the SNR of the satellite is used to detect a stay in the TBS. The proposed method can detect a higher probability TBS stay than can an existing method. 

On the other hand, the measurements during the experiments in this study were taken with the smartphone held horizontally in front of the body, which is different from the normal holding condition. When actually applying the proposed method in a logistics facility, the threshold may need to be modified to consider the actual smartphone holding condition.

In addition, three major challenges remain with our proposed method. The first is the detection accuracy of only 52.3% in the case of 1 m entry, i.e., a shallow entry angle, although this percentage is higher than that of the existing method. The presence of a satellite at a high-elevation angle is required to detect a high probability in a shallow entry angle using the proposed method. In this study, we used only GPS for our experiments. Nonetheless, it is possible to improve the selection of high-elevation satellites by adding other types of satellites, such as GLONASS, to increase the number of satellites for monitoring. Particularly in Japan, Quasi-Zenith Satellite System (QZSS) is beginning to be used to improve positioning accuracy by always having one satellite at a high-elevation angle. Since East Asia, Southeast Asia, and Australia can use QZSS due to the satellite orbits, the use of the proposed method with QZSS may be able to detect TBS stay with a high probability even at shallow entry angles [26].

The second challenge is that the proposed method can currently only be used in environments where there are no tall buildings or other buildings in the orientation of the side opening. In urban environments, where there are often buildings or other structures by the open sides of TBSs, the signals from the target satellite may already be in NLOS when the receiving device is in an outdoor area, and the SNR attenuation necessary for detection may not occur when the receiving device enters a TBS. Methods that use 3D models, such as that in [24], would enable the determination of NLOS status, but we did not use a 3D model in this study to simplify the detection of TBS stay. Since the target space in this study is a logistics facility, it is assumed that there are few cases of tall buildings in the open side orientation. However, if the applicable space is to be expanded in the future, the detection flow needs to be improved for use in urban environments.

Another challenge in the proposed method is that the degree of SNR attenuation with TBS entry may be different for each smartphone model. In this study, the threshold was set at 20% or higher attenuation compared with the outdoor space based on the results of preliminary experiments and previous studies on the attenuation of SNRs in TBSs and indoor spaces. In addition, experiments were conducted on one other phone model, and we confirmed that we could detect TBS entry even at this threshold. However, there were some cases where only one model had sufficient attenuation. It is difficult to set a threshold of SNR attenuation that can be applied to all models because the performance of the built-in GPS receiver differs for various models. The validity of these thresholds can be more precisely verified by conducting TBS entry experiments with a larger number of models.

## 7. Conclusions

In this study, we designed a method to classify satellites into four groups by using spatial characteristics of the target TBS with height, depth, and side opening orientation as parameters to identify the monitoring target satellites and achieve seamless positioning in a logistics facility that contains a TBS. Furthermore, we designed a method to detect entry into and exit from a TBS based on the change rate of the GPS SNRs of the targeted satellites. In order to confirm the effectiveness of our proposed method, we selected three locations with similar spatial characteristics to those of TBSs present in logistics facilities and conducted evaluation experiments. We set up a walking route to enter the TBS from the outside and exit again to the outside and conducted 48 measurements. The results showed that the detection accuracy of TBS stays was 65.6%. This result was about 30% higher than the existing method of detecting stay in TBS using GPS SNR. This result shows that our proposed method, which uses the spatial characteristics of TBS to select the satellites to monitor the GPS SNR changes, is more effective than the existing method. The TBS is a challenging environment that is difficult to detect accurately by conventional IO detection methods. Our proposed method is valuable because it provides a simple way to detect TBS stays without using a 3D building model.

## Figures and Tables

**Table 1:** Satellite classification for top-bounded spaces (TBS) entry detection.

Classification Group	Whether Elevation Angle Is Higher than Entry Angle Threshold	Whether Satellite Is in Side Opening Orientation
A	Yes	Yes
B	Yes	No
C	No	Yes
D	No	No

**Table 2:** SNR attenuation rate of monitoring target satellite during top-bounded spaces (TBS) entry in preliminary experiments.

Number of Measurements	SNR Attenuation Rate of Monitoring Target Satellite			
36	Average	Median	Maximum	Minimum
	−26.32%	−25.45%	−39.10%	−20.50%

**Table 3:** Comparison of detection accuracy between existing method and proposed method.

Route	Number of Times	Existing Method in [13]			Proposed Method		
		Percentage of Correct Detections	Number of Measurements Correctly Detected More than 50% of the Time per Measurement		Percentage of Correct Detections	Number of Measurements Correctly Detected More than 50% of the Time per Measurement	
1 m entry	18	27.1%	5	27.8%	52.3%	10	55.6%
2 m entry	18	43.9%	7	38.9%	60.0%	14	77.8%
3 m entry	12	35.9%	2	16.7%	85.8%	12	100.0%
Total	48	35.6%	14	29.2%	65.6%	36	75.0%

**Table 4:** Comparison of detection accuracy between existing method and proposed method.

		Model A			Model B		
Model Name		Fujitsu Arrows m04			Google Pixel3a		
OS Version		Android 7.1.1			Android 10		
Route	Number of times	Percentage of correct detections	Number of measurements correctly detected more than 50% of the time per measurement		Percentage of correct detections	Number of measurements correctly detected more than 50% of the time per measurement	
1 m entry	3	100.0%	3	100.0%	79.2%	3	100.0%
2 m entry	3	33.3%	1	33.3%	69.2%	3	100.0%
3 m entry	3	88.1%	3	100.0%	82.1%	3	100.0%
Total	9	73.9%	7	77.8%	76.9%	9	100.0%
